# Integrated methylome and transcriptome analysis provides insight into DNA methylation-mediated networks in sexual dimorphism of *Vernicia montana*

**DOI:** 10.1186/s12870-026-09376-y

**Published:** 2026-06-26

**Authors:** Xiang Dong, Lin Zhang, Huaguo Zhu, Jinping Peng, Congchuan Lu, Wenying Li

**Affiliations:** 1 College of Biology and Agricultural Resources, Huanggang Normal University, Huanggang, Hubei 438000 China; 2 Economic Crop Research Institute, Hubei Academy of Agricultural Sciences, Wuhan, Hubei 430000 China; 3 State Key Laboratory of Woody Oil Resources Utilization, Central South University of Forestry and Technology, Changsha, 410004 China; 4 Hubei Key Laboratory of Economic Forest Germplasm Improvement and Resources Comprehensive Utilization, Huanggang, Hubei 438000 China

**Keywords:** *Vernicia montana*, Sexual dimorphism, DNA methylation, Differentially methylated and expressed transcription factor, Co-expression network

## Abstract

**Background:**

Sexual dimorphism is fundamental to reproduction in dioecious plants and is regulated by both genetic and epigenetic mechanisms. DNA methylation is a central epigenetic mark known to influence phenotypic variation in plants. However, its specific role in shaping sexual dimorphism in dioecious trees remains poorly understood. To address this question, we performed integrated genome-wide DNA methylome and transcriptome analyses of four tissue types in the dioecious tung tree (*Vernicia montana*), including male and female flower buds and their corresponding leaves.

**Results:**

Our analysis revealed distinct DNA methylation patterns between male and female tissues. Notably, the coordination between DNA methylation reprogramming and transcriptional regulation appeared to be more strongly associated with reproductive development than with vegetative growth in *V. montana*. We identified a set of sex-biased genes that may reflect different reproductive strategies between the sexes. Further analysis identified several key transcription factors (TFs) potentially associated with promoter differentially methylated regions (DMRs), including flowering-time regulators (e.g., *FRS5*, *REM16*, and *VRN1*) and TFs involved in hormone signaling pathways such as jasmonic acid, auxin, and salicylic acid signaling. Cis-regulatory element analysis showed that some promoter DMRs overlapped with hormone response elements related to abscisic acid, auxin, and gibberellin. Co-expression network analysis further revealed potential regulatory correlations among promoter DMR-mediated TFs, hormone-responsive pathways, and key floral development regulators.

**Conclusions:**

Collectively, our results suggest that interactions among DNA methylation, transcriptional regulation, and hormone-responsive pathways may contribute to the establishment of sexual dimorphism in *V. montana*. This study provides the first integrated view of these regulatory layers in *V. montana* and supports a species-specific regulatory framework for understanding the epigenetic basis of sexual dimorphism in this economically important dioecious tree. The proposed framework is based on multi-omics analyses and warrants further validation through targeted functional studies.

**Supplementary Information:**

The online version contains supplementary material available at https://doi.org/10.1186/s12870-026-09376-y.

## Introduction

Among flowering plants, approximately 6% of species in 43% of all angiosperm families are dioecious, with female and male flowers developing on separate individuals [1–3]. The differences in both sexual features and secondary sex characteristics, including morphological, physiological, resistance and other life history traits between male and female individuals are called sexual dimorphism [4, 5]. Although generally less pronounced in plants than in many animal groups, sexual dimorphism occurs to varying degrees across numerous dioecious species [4, 6]. For example, in *Trichosanthes pilosa*, male flowers are arranged in racemes, flower early, and are caducous, whereas female flowers are solitary, flower later, and are long-lived [7].

Sexual dimorphism often evolves during the transition from hermaphroditism to dioecy, a shift that allows sex specific traits to be optimized for fitness [8, 9]. Research on the relationship between dimorphism and sex chromosomes indicates that sexually dimorphic phenotypes are frequently associated with genes located on sex chromosomes, which often exhibit sex-biased transmission and genomic distributions [10, 11]. Moreover, sex-linked genes likely contribute more substantially to dimorphism than genes elsewhere in the genome [12, 13]. This is exemplified by sequence divergence between homologous haplotypes within sex determining regions, a divergence driven by sexually antagonistic selection that can directly produce dimorphic traits [14–16]. Studies in poplar, willow and papaya have identified functions for genes with sex-biased or sex-limited expression in shaping sexual dimorphism [17–19]. Consequently, investigating sexual dimorphism can significantly advance our understanding of sex differentiation and determination mechanisms and aid in identifying key genes within sex specific regulatory networks.

The evolution of unisexual flowers involves multiple developmental mechanisms, including floral primordia suppression, programmed organ abortion, and epigenetic regulation [20]. Among these, DNA methylation serves as a central epigenetic mark and plays a critical role in flower development, plant sex differentiation, and even sex determination [21–23]. In plants, DNA methylation occurs in CG, CHG, and CHH (where H is A, T, or C) and is closely linked with histone H3K9me modification [24, 25]. Methylation in CG contexts is commonly found in gene bodies and promoter regions, whereas non-CG (CHG and CHH) methylation predominates in transposable elements [26]. *De novo* methylation is largely directed by the RNA-directed DNA methylation (RdDM) pathway, which involves small interfering RNAs, scaffold RNAs, and various protein cofactors [27]. Accumulating evidence indicates that DNA methylation is closely associated with sex differentiation in diverse dioecious plants, where epigenetic alterations in key floral developmental genes can directly modulate organ identity [28]. For example, in hexaploid persimmon (*Diospyros kaki*), methylation of the *MeGI* promoter in male flowers activates a small RNA (*smMeGI*), which suppresses *MeGI* expression and promotes stamen development [29]. Likewise, early flowering in male papaya has been linked to epigenetic regulation of the autosomal genes *SVP* and *APETALA1* [30]. To date, few studies have focused on the variation in secondary sexual dimorphism in flowers and leaves, and its underlying DNA methylation regulation.

*Vernicia montana*, a dioecious tung tree, is an important woody-oil tree species cultivated in regions including East Asia, Southeast Asia, North and South America, and Australia [31]. Its oil is used in the production of eco-friendly coatings, printing inks, plasticizers, pharmaceuticals, chemical reagents, resins, and biodiesel [32, 33]. Clarifying the mechanisms of sex differentiation and determination in *V. montana* is important for the tung oil industry, as oil production depends entirely on female trees.

The sex of *V. montana* is likely determined by a ZZ/ZW chromosome system [34]. Previous studies have showed clear differences between male and female flowers in morphology, phenology, gene expression, and hormone levels [35]. For example, female flowers contain a ring of aborted stamens, while male flowers lack pistils entirely, and male bud differentiation starts about nine months earlier than in females. Although sex-biased genes and phytohormone pathways have been implicated in this process [35], the role of DNA methylation in regulating sexual dimorphism in *V. montana* remains poorly understood.

Therefore, we integrated DNA methylome and transcriptome profiles from male and female flower buds and leaves. This allowed us to systematically compare genome-wide DNA methylation patterns and gene expression between sexes and to identify potential DNA methylation-mediated transcription factors (TFs) associated with sexual dimorphism, thereby contributing to the understanding of molecular mechanisms in plant sex differentiation.

## Materials and methods

### Plant materials

**Fig. 1:**
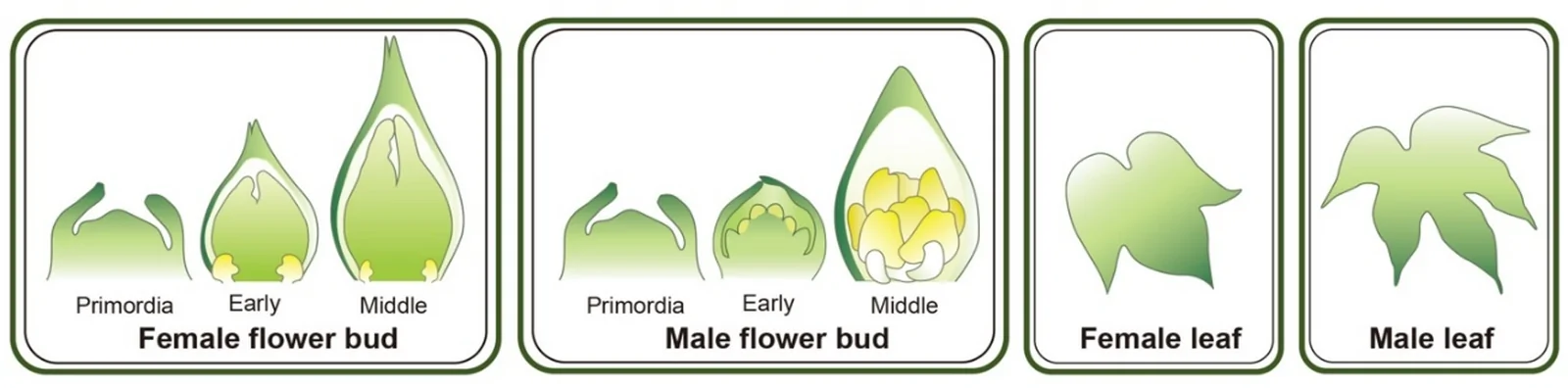
Flower buds and leaves used for transcriptome and DNA methylome sequencing

The plant materials used in this study were sampled from wild *V. montana* trees at the Jiufeng Experimental Forest Farm. Between April and September 2024, female and male flower buds at three developmental stages (primordia, early, and middle), which correspond to stages F1, F4, and F5 in females and M1, M2, and M3 in males as defined by Li et al. [35] (Fig. 1). These stages represent pivotal phases of sex differentiation. Mature leaves were also collected. To obtain representative transcriptional and epigenetic states, floral samples of each sex were pooled across collection dates for subsequent transcriptomic and DNA methylome sequencing. All collected tissues were immediately frozen in liquid nitrogen and stored at -80 °C until subsequent analysis. Three biological replicates were prepared for each tissue type.

### Identification of sex-biased genes and GO enrichment analysis

Total RNA was extracted from different tissues using the Polysaccharide Polyphenol Plant Total RNA Extraction Kit (TIANGEN). mRNA libraries were constructed with the Hieff NGS^®^ Ultima Dual-mode mRNA Library Prep Kit according to the manufacturer’s instructions. Sequencing was performed on the DNBSEQ-T7 platform with 150 bp paired-end reads. Raw reads were processed using fastp [36] to obtain high-quality clean data. Ribosomal RNA sequences were identified and removed by aligning reads to a ribosomal database using bowtie2 [37]. The remaining reads were aligned to the haploid female *V. montana* reference genome [38] using HISAT2 [39]. Gene expression levels were quantified using StringTie [40] and normalized by the Transcripts Per Million (TPM) method. Differentially expressed genes (DEGs) were identified using DESeq2 in R. Genes significantly upregulated (log2foldChange > 1 and P-value < 0.05) in female flower buds or leaves were classified as female-biased genes, while those upregulated in male tissues were defined as male-biased genes. Gene Ontology (GO) and Kyoto Encyclopedia of Genes and Genomes (KEGG) enrichment analysis were both conducted using the Omicshare platform (https://www.omicshare.com/tools/).

### Whole-genome bisulfite sequencing (WGBS) and DNA methylome analysis

Genomic DNA was isolated from tissues using the CTAB method [41]. DNA concentration and integrity were assessed using a Qubit Fluorometer (Invitrogen) and agarose gel electrophoresis, respectively. For each sample, 200 ng of genomic DNA was spiked with λ DNA as an internal control for bisulfite conversion and fragmented to 200–400 bp using a Covaris M220 ultrasonicator. Library construction included end repair, dA-tailing, and adapter ligation, followed by bisulfite conversion with the EZ DNA Methylation-Gold™ Kit (Zymo Research). Converted DNA was amplified by PCR and sequenced on the Illumina NovaSeq 6000 platform with 150 bp paired end reads. Raw bisulfite sequencing data were processed with Trimmomatic [42] for quality control. Clean reads were aligned to the haploid *V. montana* genome using BSMAP [43], and differentially methylated regions (DMRs) were identified using metilene [44].

### Integrated analysis of transcriptome and DNA methylome

DEGs overlapping with DMRs in either the gene body region or within 2 kb upstream of the gene were considered as DEGs potentially regulated by DNA methylation [45]. So, these DEGs were defined as DMR-mediated genes in this study. Among these genes, TFs were identified using the PlantTFDB (https://planttfdb.gao-lab.org/).

### Promoter cis-element analysis

Putative promoter sequences, defined as the 2 kb regions upstream of the start codon (ATG), were extracted for each candidate gene. Subsequently, cis-regulatory elements within these sequences were predicted using the PlantCARE database (http://bioinformatics.psb.ugent.be/webtools/plantcare/html/).

### Weighted gene co-expression network analysis (WGCNA)

Weighted gene co-expression networks were constructed using the WGCNA package in R, based on transcriptome data from female and male flower buds and leaves of *V. montana*. The analysis followed a standardized pipeline comprising: (1) data normalization to ensure suitability for network construction, (2) selection of an appropriate soft-thresholding power to achieve approximate scale-free topology, (3) identification of co-expression modules through hierarchical clustering, (4) evaluation of module-trait associations to detect sex-related modules, and (5) selection of hub genes based on module membership (MM) and gene significance (GS) with the screening criteria of |MM| > 0.8 and |GS|> 0.2. The resulting co-expression network was visualized in Cytoscape to illustrate regulatory relationships.

## Results

### Identification and functional enrichment of sex-biased genes

**Fig. 2:**
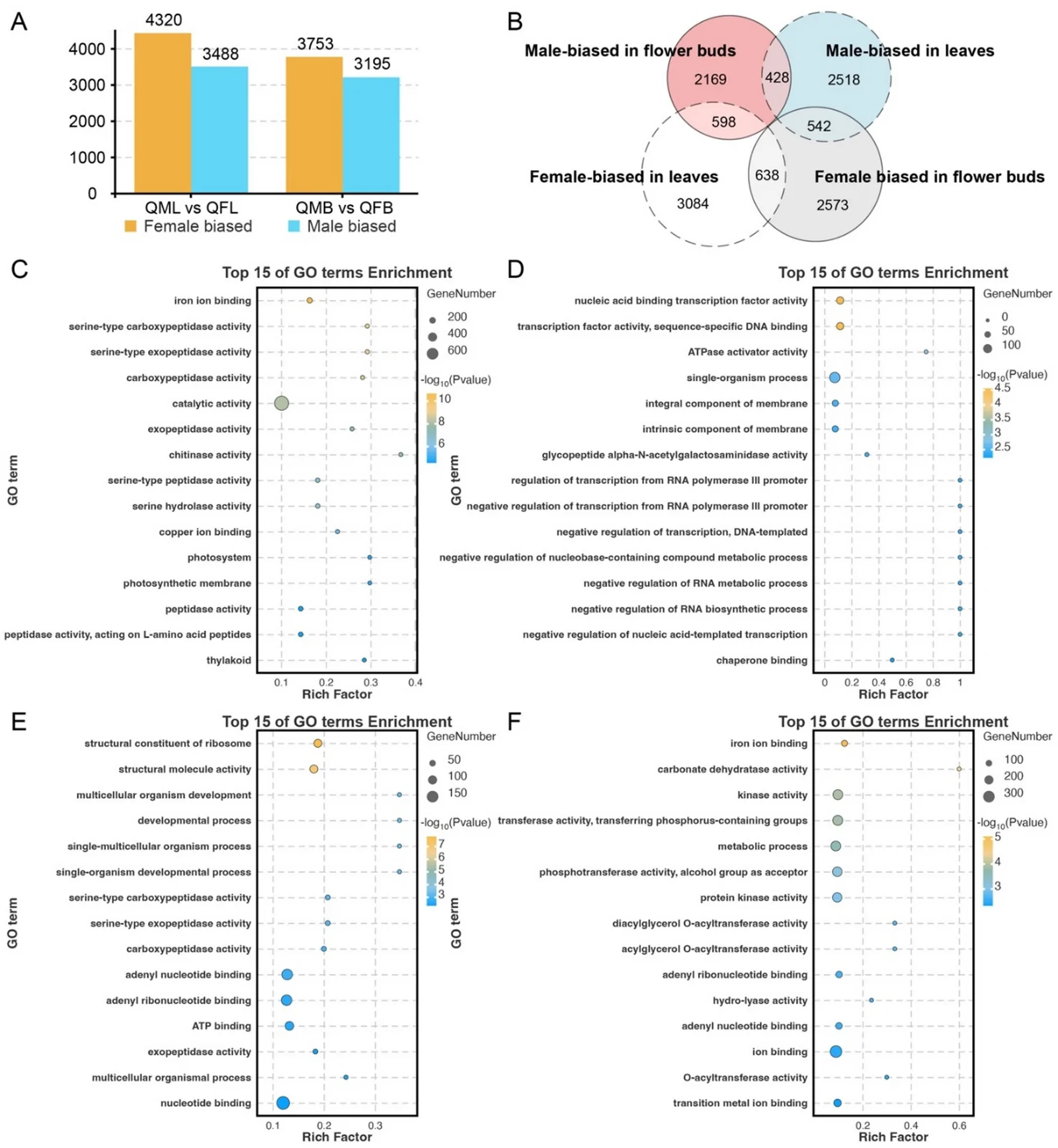
Identification and functional enrichment of sex-biased genes in *V. montana.* **A** Bar plot showing the number of sex-biased genes in male (QMB) and female (QFB) flower buds, and in male (QML) and female (QFL) leaves. **B** Venn diagram illustrating the overlap of sex-biased genes across different tissues. **C**-**F** GO enrichment analysis of female-biased genes in flower buds (**C**), male-biased genes in flower buds (**D**), female-biased genes in leaves (**E**), and male-biased genes in leaves (**F**)

Comparative transcriptome analysis revealed 6,948 DEGs when comparing male (QMB) to female (QFB) flower buds, with 3,753 genes showing female-biased expression and 3,195 genes showing male-biased expression. In leaves, 7,808 DEGs were identified in female (QFL) versus male (QML) samples, comprising 4,320 female-biased and 3,488 male-biased genes (Fig. 2A). Among these, 638 genes showed consistently higher expression across both female tissues (QFB and QFL), while 428 genes were consistently upregulated in male tissues (QMB and QML) (Fig. 2B).

GO enrichment analysis indicated that female-biased genes in flower buds were significantly associated with metabolic and energy-related process (e.g., catalytic activity, photosystem, photosynthetic membrane), activities crucial for hormone regulation (e.g., carboxypeptidase activity, exopeptidase activity), as well as defense responses (e.g., iron/copper ion binding, chitinase activity) (Fig. 2C). In contrast, male-biased genes in flower buds were enriched for functions related to transcription factor activity and transcriptional regulation related processes (e.g., regulation / negative regulation of transcription/RNA metabolic process, RNA polymerase III-related processes) (Fig. 2D).

GO enrichment of male- and female-biased genes in leaf tissues also showed divergent profiles. Female-biased genes were primarily linked to protein synthesis (e.g., structural constituent of ribosome and structural molecule activity), development and tissue construction (e.g., multicellular organism development and developmental processes), energy metabolism (e.g., adenyl nucleotide binding and ATP binding), and hormone regulation (e.g., carboxypeptidase activity) (Fig. 2E). Whereas male-biased genes were more involved in signal transduction and regulation (e.g., kinase activity, protein kinase activity, and phosphotransferase activity), photosynthesis and carbon metabolism (e.g., carbonate dehydratase activity), lipid metabolism and storage (e.g., diacylglycerol O-acyltransferase activity), and defense responses (e.g., iron ion binding and transition metal ion binding) (Fig. 2F).

### Identification and genomic distribution analysis of DMRs

We detected 24,911 CG-type, 26,796 CHG-type, and 413,670 CHH-type DMRs in QMB versus QFB. Hypermethylated DMRs (HDMRs) in each sequence context exhibited a sex-biased distribution: for CG-type, 11,214 were in QFB versus 13,697 in QMB; for CHG-type, 7,331 in QFB versus 19,465 in QMB; and for CHH-type, 2,026 in QFB versus 411,644 in QMB (Figure S1). Overall, QFB contained fewer HDMRs than QMB. A similar trend was observed when comparing QML to QFL, where, for example, CG-type HDMRs numbered 683 in QFL versus 747 in QML, while CHG-type HDMRs were 1,307 in QFL versus 1,429 in QML (Figure S1). Notably, the difference in DMR numbers in flower buds (QMB vs. QFB) was greater than that in leaves (QML vs. QFL).

**Fig. 3:**
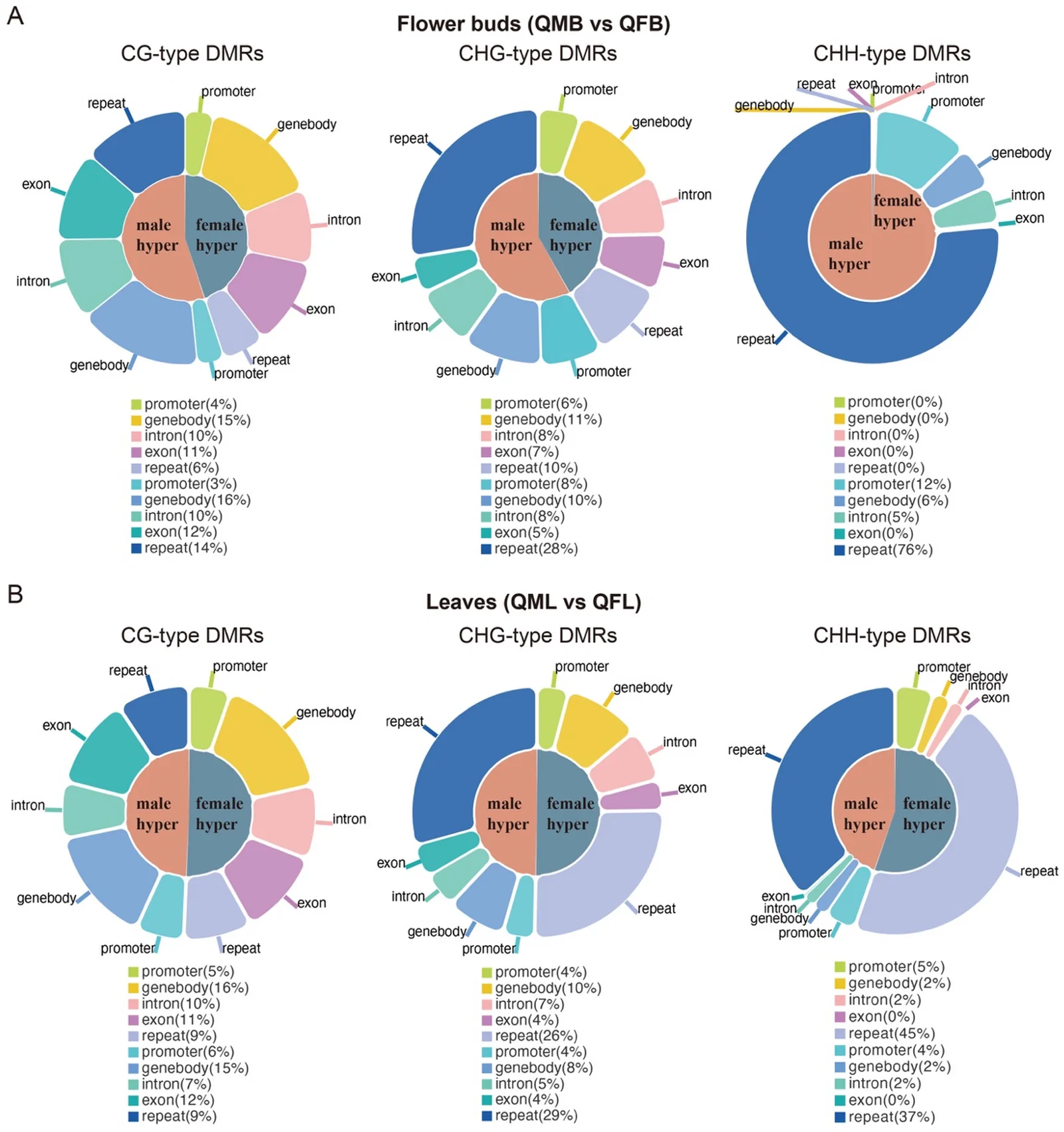
Genomic distribution of hypermethylated DMRs (HDMRs) across the *V. montana* genome in flower buds (**A**) and leaves (**B**) . **A** Distribution of CG-, CHG-, and CHH-type HDMRs between QMB and QFB. **B** Distribution of the three HDMR types between QML and QFL

Regarding distribution patterns, CG-type HDMRs displayed a similar pattern in both QMB and QFB, being predominantly enriched in gene body regions and less frequent in promoter regions. Similarly, CHH-type HDMRs were mainly located in repetitive sequences in both sexes, with fewer occurrences in exon regions (Fig. 3A). In contrast, the distribution of CHG-type HDMRs differed markedly in QMB versus QFB: in QFB, they were primarily enriched in gene bodies, followed by repetitive sequences, whereas in QMB, they were mainly concentrated in repetitive sequences, followed by gene bodies (Fig. 3A). The distribution patterns of all three HDMR types when comparing QML to QFL were generally consistent with those observed in flower buds (Fig. 3B).

### Identification and functional enrichment of DMR-mediated sex-biased genes

An integrated analysis of transcriptome and DNA methylome data from *V. montana* flower buds and leaves was performed. A total of 4,595 potentially DMR-mediated DEGs were identified in flower buds (QMB versus QFB), of which 2,547 were female-biased and 2,048 were male-biased (Figure S2). When comparing QML to QFL, 694 DMR-mediated DEGs were detected, consisting of 403 female-biased and 291 male-biased (Figure S2).

**Fig. 4:**
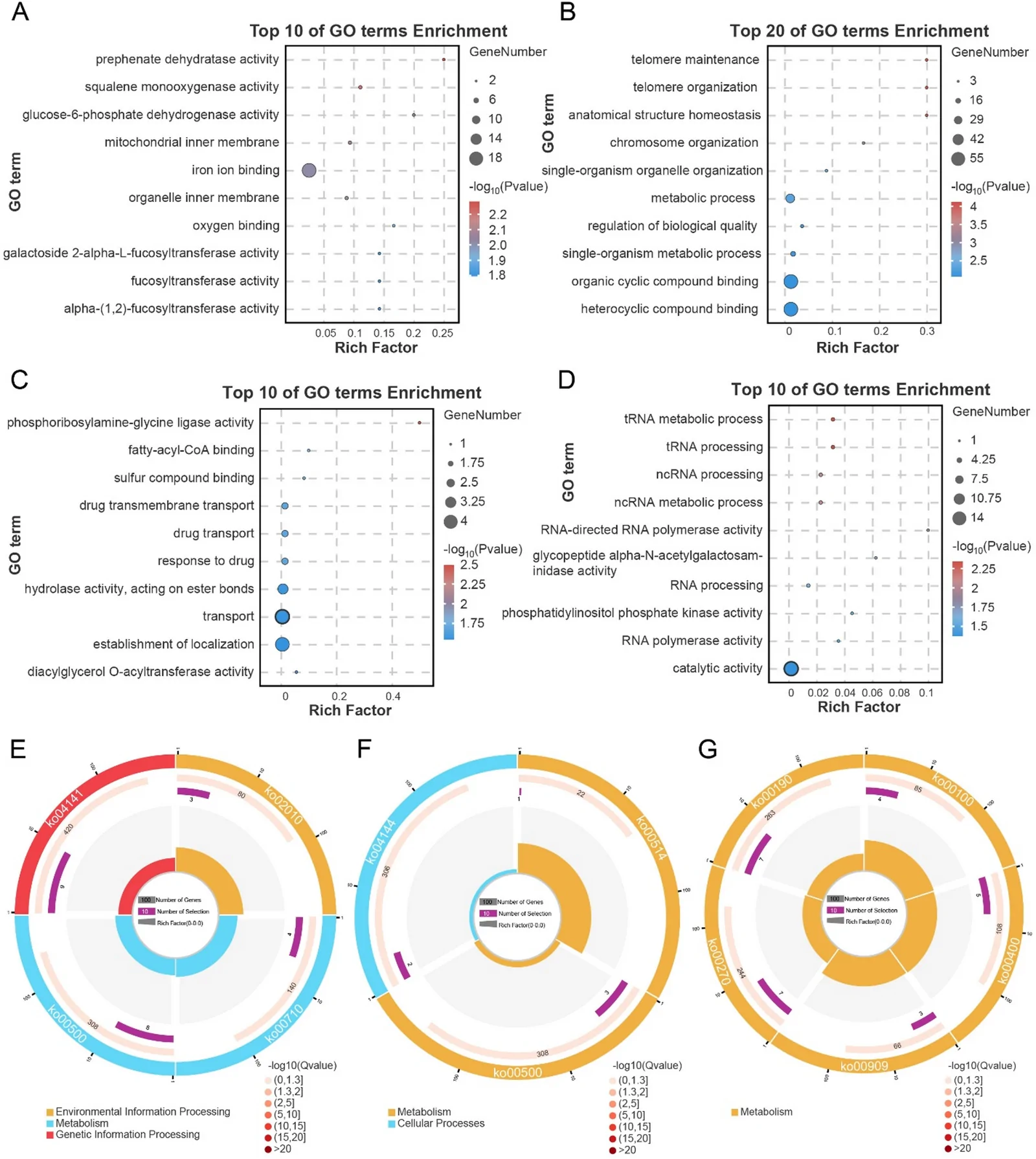
GO and KEGG enrichment analysis of DMR-mediated DEGs. The top 10 enriched GO terms for female- and male-biased genes were shown as bubble plots for flower buds (**A**, **B**) and leaves (**C**, **D**), respectively. Significantly enriched KEGG pathways for male-biased genes in flower buds (**E**) and leaves (**F**), and for female-biased genes in flower buds (**G**) were displayed in circle diagrams. In the KEGG circle plots, the outer colored sectors represented KEGG functional categories, including Environmental Information Processing, Metabolism, Genetic Information Processing, and Cellular Processes. The light-colored inner bars indicated pathway enrichment significance based on -log10(Qvalue), with darker colors representing higher significance. Purple bars represented the number of enriched genes in each pathway, and the central sectors indicated the rich factor of each pathway

GO annotation showed that among these DMR-mediated sex-biased genes in flower buds, female-biased genes were significantly enriched in energy supply (e.g., mitochondrial / organelle inner membrane) and molecular functions associated with defense responses (e.g., prephenate dehydratase activity, glucose-6-phosphate dehydrogenase activity and iron ion binding) (Fig. 4A). In contrast, male-biased genes were significantly enriched in biological processes related to maintaining genetic material stability, including telomere maintenance/organization and chromosome organization (Fig. 4B). In leaves, DMR-mediated female-biased genes were significantly enriched in processes related to lipid synthesis, metabolism, and substance transport, such as transport, fatty-acyl-CoA binding, and acyltransferase activity (Fig. 4C). Meanwhile, male-biased genes were significantly enriched in tRNA/ncRNA metabolic and modification processes (Fig. 4D).

KEGG enrichment analysis revealed that DMR-mediated male-biased genes in both flower buds and leaves were significantly enriched in Starch and sucrose metabolism (ko00500) (Fig. 4E and F). On the other hand, female-biased genes in flower buds were enriched in pathways related to phytohormone synthesis [e.g., phenylalanine, tyrosine and tryptophan biosynthesis (ko00400); steroid biosynthesis (ko00100)] and defense responses [e.g., cysteine and methionine metabolism (ko00270); oxidative phosphorylation (ko00190); sesquiterpenoid and triterpenoid biosynthesis (ko00909) (Fig. 4G)]. Not any significantly enriched pathway was identified in female-biased genes in flower leaves.

### Function annotation and expression profiles of promoter DMR-mediated TFs

**Fig. 5:**
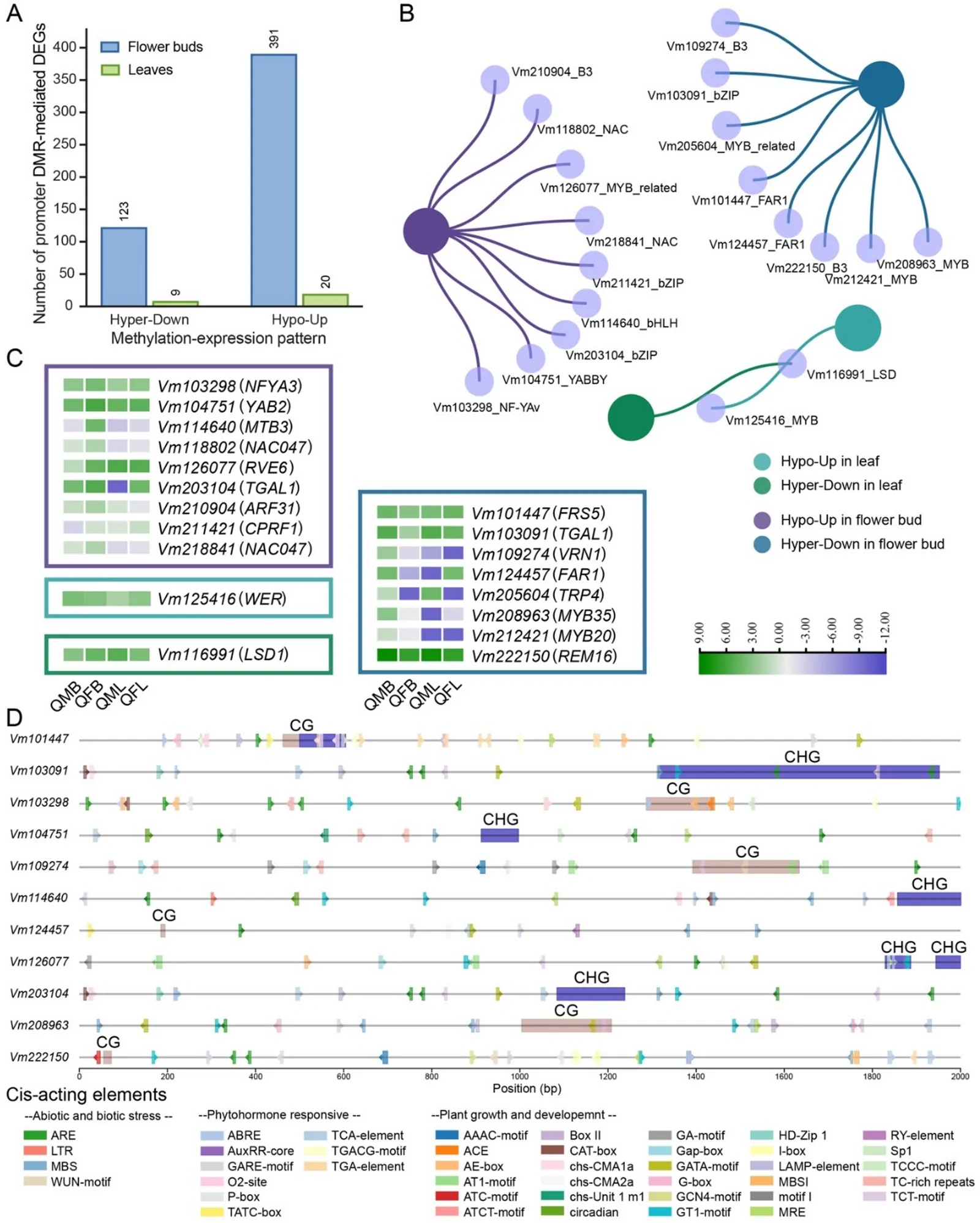
Expression profiles of promoter DMR-mediated TFs in *V. montana.* **A** Bar chart showing the number of promoter DMR-mediated DEGs in flowers and leaves. **B** Methylation-expression correlation patterns of the promoter DMR-mediated TFs. **C** Heatmap of expression profiles for the promoter DMR-mediated TFs across different tissues. **D** Promoter cis-acting elements and differential methylome profiles

Based on the established role of DNA methylation in transcription repression [46], we further examined the methylation patterns in the promoter regions of DMR-mediated DEGs to explore this regulatory relationship. A total of 514 DMR-mediated DEGs exhibited promoter hypermethylation-mediated downregulated (hyper-down) or hypomethylation-mediated upregulated (hypo-up) expression profiles in QMB versus QFB. This set included 123 genes with hyper-down expression, and 391 genes showed hypo-up expression (Fig. 5A). Similarly, in QML versus QFL, 9 hyper-down and 20 hypo-up genes were identified (Fig. 5A). Among these DEGs, 19 were TFs, including 8 hyper-down and 9 hypo-up in QMB versus QFB, and 1 hyper-down and 1 hypo-up in QML versus QFL (Fig. 5B). Based on the criteria for sex-biased expression, where genes with higher expression in male tissues are defined as male-biased and those with higher expression in female tissues are defined as female-biased, the hyper-down methylation pattern aligned with male-biased expression, while the hypo-up pattern aligned with female-biased expression.

Functional annotation of these 19 potentially promoter DMR-mediated TFs showed that 7 are potentially involved in reproductive processes (e.g., flower organ development, flowering time), 4 in hormone signaling, 4 stress responses, and the remaining 4 in the regulation of plant morphology and physiology (Table 1). Among them, 11 TFs showed relatively high expression levels (TPM > 5) and belonged to multiple TF families, including *Vm208963* (*MYB35*) and *Vm126077* (*RVE6*) from the MYB family, *Vm109274* (*VRN1*) and *Vm222150* (*REM16*) from the B3 family, *Vm124457* (*FAR1*) and *Vm101447* (*FRS5*) from the FAR1 family, *Vm103091* (*TGAL1*) and *Vm203104* (*TGAL1*) from the bZIP family, as well as *Vm104751* (*YAB2*), *Vm103298* (*NF-YA3*), and *Vm114640* (*MTB3*) from the YABBY, NF-YA, and bHLH families, respectively (Fig. 5C; Table 1). Among them, *Vm104751*, *Vm103298*, and *Vm103091* are located on the Z chromosome (ChrA02), while *Vm203104* and *Vm205604* are located on the W chromosome (ChrB02; Table 1).

**Table 1:** Functional annotation of potentially promoter DMR-mediated TFs

Gene ID	Homolog	Chr	TF Family	Expression Bias	Function Prediction	Class
*Vm101447*	*FRS5*	ChrA01	FAR1	Male-biased in buds	Regulates flowering time [47].	Reproductive processes
*Vm109274*	*VRN1*	ChrA04	B3	Male-biased in buds	Regulates flowering time [48].	Reproductive processes
*Vm124457*	*FAR1*	ChrA10	FAR1	Male-biased in buds	Regulates flowering time [49].	Reproductive processes
*Vm126077*	*RVE6*	ChrA10	MYB_related	Female-biased in buds	Regulates flowering time, coordinates leaf growth, and antagonistically regulates hypocotyl elongation [50].	Reproductive processes
*Vm222150*	*REM16*	ChrB09	B3	Male-biased in buds	Regulates flowering time [51].	Reproductive processes
*Vm208963*	*MYB35*	ChrB04	MYB	Male-biased in buds	Acts in tapetal cells during early flower development [52].	Reproductive processes
*Vm210904*	*ARF31*	ChrB04	B3	Female-biased in buds	Influences petal development and shoot growth [53].	Reproductive processes
*Vm103298*	*NF-YA3*	ChrA02	NF-YA	Female-biased in buds	Modulates auxin signaling in early-stage embryos.	Hormone signaling
*Vm103091*	*TGAL1*	ChrA02	bZIP	Male-biased in buds	Participates in immune [54] responses through the salicylic acid signaling pathway [55].	Hormone signaling
*Vm203104*	*TGAL1*	ChrB02	bZIP	Female-biased in buds	Participates in immune responses through the salicylic acid signaling pathway [55].	Hormone signaling
*Vm114640*	*MTB3*	ChrA06	bHLH	Female-biased in buds	Negatively regulates jasmonic acid-mediated transcriptional responses [56].	Hormone signaling
*Vm116991*	*LSD1*	ChrA07	LSD	Male-biased in leaves	Modulates cell death triggered by cold stress [57].	Stress responses
*Vm118802*	*NAC047*	ChrA07	NAC	Female-biased in buds	Enhances tolerance to multiple abiotic stresses [58].	Stress responses
*Vm218841*	*NAC047*	ChrB07	NAC	Female-biased in buds	Enhances tolerance to multiple abiotic stresses [58].	Stress responses
*Vm212421*	*MYB20*	ChrB05	MYB	Male-biased in buds	Negatively regulates tolerance to salt and cold stress [59].	Stress responses
*Vm104751*	*YAB2*	ChrA02	YABBY	Female-biased in buds	Controls abaxial cell fate determination [60].	Others
*Vm125416*	*WER*	ChrA10	MYB	Female-biased in leaves	Regulates fate of root-hair cells [61].	Others
*Vm205604*	*TRP4*	ChrB02	MYB_related	Male-biased in buds	Regulates the transcription of hundreds of genes involved in developmental responses to environmental cues [62].	Others
*Vm211421*	*CPRF1*	ChrB05	bZIP	Female-biased in buds	Regulates chloroplast development and leaf coloration [63].	Others

Notably, flowering time-related TFs showed distinct sex-specific expression profiles (Fig. 5C; Table 1). For example, female-biased *Vm126077* (*RVE6*) was expressed approximately 12-fold higher in female flower buds than in male buds. While *Vm101447* (*FRS5*) and *Vm222150* (*REM16*) were expressed in both sexes, their transcript levels in male flower buds were about 2-fold and 4-fold higher, respectively. *Vm109274* (*VRN1*) and *Vm124457* (*FAR1*) only expressed in male flower buds. Moreover, *Vm208963* (*MYB35*), which regulates early tapetum development, was exclusively expressed in male flower buds, whereas *Vm114640* (*MTB3*), involved in jasmonic acid response, was specific to female flower buds. Meanwhile, *Vm104751* (*YAB2*), involved in abaxial cell fate determination, and *Vm103298* (*NF-YA3*), influencing auxin response during early embryogenesis, both showed significantly higher expression in female flower buds. Interestingly, the salicylic acid-responsive alleles *Vm203104* (*TGAL1*) and *Vm103091* (*TGAL1*) were upregulated in female (2-fold) and male (9-fold) flower buds, respectively.

We further examined whether DMRs spatially overlapped with cis-regulatory elements in the promoters of the 11 promoter DMR-mediated TFs that showed relatively high expression levels. Among these TFs, 6 TFs contained CG- and/or CHG-type DMRs within their promoter regions, which spatially overlapped with 3 to 4 cis-elements involved in plant growth, hormone signaling, and stress responses (Fig. 5D). For example, CHG-type DMRs covered TGA-element (an auxin-responsive element), chs-CMA1a (part of a light responsive element), and GARE-motif (a gibberellin-responsive element) in *Vm101447*; the MBS (a MYB binding site involved in drought-inducibility), GT1-motif (light responsive element), TCT-motif (part of a light responsive element), and ARE (an anaerobic induction-essential element) in *Vm103091*; and Sp1 (a light responsive element), ABRE (an abscisic acid-responsive element), AuxRR-core (an auxin-responsive element), and GT1-motif in *Vm126077*. In contrast, CG-type DMRs covered the ABRE, AE-box (part of a light-responsive module), and ACE (light responsive element) in *Vm103298*; the TCT-motif, WUN-motif (a wound-responsive element), and AT1-motif (part of a light responsive element) in *Vm109274*; and the GATA-motif (part of a light-responsive element), ABRE, and G-box (a light-responsive element) in *Vm208963*. Notably, the majority of the cis-elements overlapped by DMRs were light-responsive elements. Hormone-related elements, especially the abscisic acid-responsive element (ABRE), were also present in most (4 out of 6) of the TFs.

### Construction of weighted gene co-expression network

**Fig. 6:**
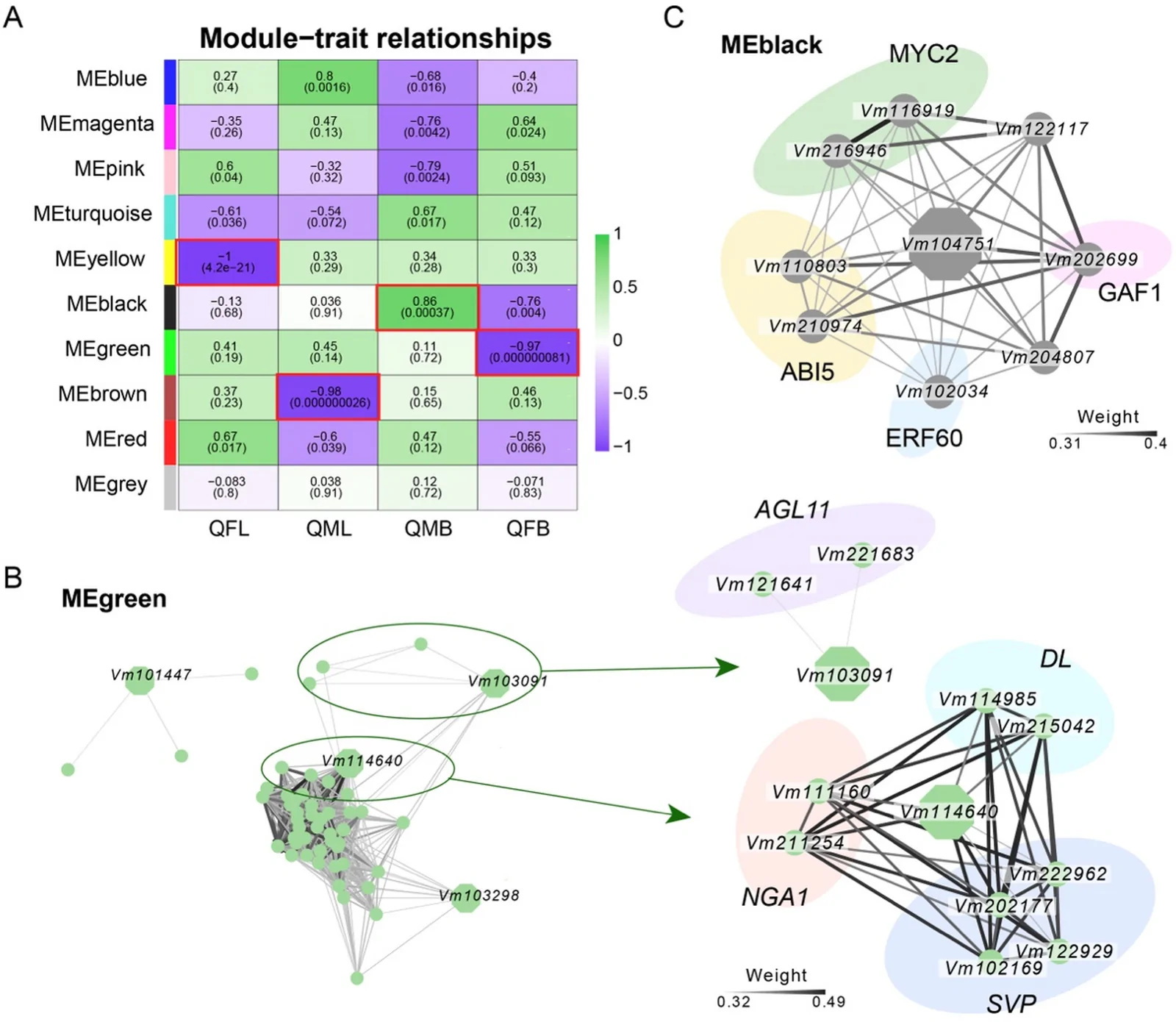
Identification and network analysis of trait-associated modules in *V. montana*. **A** Heatmap depicting module-trait relationships across all identified co-expression modules. **B** and **C** Co-expression subnetworks of hub TFs for the green (**B**) and black (**C**) modules

We performed WGCNA to explore co-expression networks underlying sexual dimorphism in *V. montana*. This analysis identified ten distinct co-expression modules, with sizes ranging from 40 to 6,583 genes (Fig. 6A). Module-trait relationship analysis revealed several strong and significant associations with QML, QFL, QMB, and QFB. Specifically, the ME-yellow (1,871 genes), the ME-brown (3,620 genes), the ME-green (1,434 genes), and ME-black (648 genes) were selected as the modules with the largest correlation coefficient with QFL (*r* = -1, *p* = 4.2 × 10⁻²¹), QML (*r* = -0.98, *p* = 2.6 × 10⁻⁸), QFB (*r* = -0.97, *p* = 8.1 × 10⁻⁸), and QMB (*r* = -0.86, *p* = 3.7 × 10⁻⁴), respectively.

GO enrichment analysis on genes from yellow, brown, green, and black modules gain insight into biological significance of these genes. Genes in the yellow module were significantly enriched in for molecular functions related to nucleotide binding, including adenyl ribonucleotide binding, ATP binding, and purine ribonucleoside triphosphate binding (Figure S3A). The brown module was predominantly enriched for terms associated with translation, specifically structural constituent of ribosome and structural molecule activity (Figure S3B). In the green module, genes were significantly enriched in biological processes such as response to metal ion, response to inorganic substance, and transcription export complex (Figure S3C). For the black module, significant enrichment was observed in diverse biological processes including photosynthesis, meiotic cell cycle and amino acid biosynthetic process (Figure S3D).

Hub genes were identified from each module using screening criteria of |MM| > 0.8 and |GS| > 0.2. A total of 363 hub TFs were identified, distributed across the yellow (77), brown (138), green (95), and black (53) modules. These hub TFs belonged to 46 families, with prominent representation from bHLH, MYB, C2H2, and WRKY families (Table S1). Among the previously described set of promoter DMR-mediated TFs with relatively high expression levels, several were identified as hub TFs within their respective modules: *Vm101447*, *Vm103091*, *Vm103298*, and *Vm114640* in the green module, *Vm203104* in the brown module, and *Vm104751* in the black module.

As floral organs represent the most direct and fundamental manifestation of sexual dimorphism in dioecious plants and serve as the key tissue for studying sex determination, this study subsequently focused on an in-depth analysis of the green and black modules, which showed the strongest significant correlations with QMB and QFB, respectively. To further decipher the molecular basis of sexual dimorphism in *V. montana* from an epigenetic regulatory perspective, we mapped previously identified sex-biased promoter DMR-mediated TFs with relatively high expression in flower buds onto the green and black co-expression networks. This mapping aimed to elucidate how DNA methylation may drive developmental differences between male and female flowers by regulating core transcriptional regulators.

In the green module, the promoter DMR-mediated TFs *Vm101447*, *Vm103091*, *Vm103298*, and *Vm114640* were co-expressed with 3, 8, 11, and 38 hub TFs, respectively, and exhibited significant co-expression relationships (edge weight > 0.3) with multiple hub TFs involved in flower development (Fig. 6B). For instance, *Vm114640* was co-expressed with the flowering repressor *Short Vegetative Phase* (*SVP*; *Vm102169*, *Vm122929*, *Vm202177*, *Vm222962*), the lateral organ development and ABA synthesis-related factor *NGATHA1* (*NGA1*; *Vm111160*, *Vm211254*), and the floral organ identity determinant *DROOPING LEAF* (*DL*; *Vm114985*, *Vm215042*). *Vm103091* was co-expressed with the MADS-box gene *AGAMOUS-LIKE 11* (*AGL11*; *Vm121641*, *Vm221683*).

In the black module, *Vm104751* was co-expressed with eight other hub TFs with strong associations, six of which are associated with hormone response and signaling (Fig. 6C). These included the ethylene-responsive factor *Ethylene Response Factor 60* (*ERF60*; *Vm102034*), the gibberellin signaling regulator *GAI-ASSOCIATED FACTOR 1* (*GAF1*; *Vm202699*), the abscisic acid-responsive factor *ABSCISIC ACID-INSENSITIVE 5* (*ABI5*; *Vm110803*, *Vm210974*), and the jasmonate-regulated factor *Myelocytomatosis protein 2* (*MYC2*; *Vm116919*, *Vm216946*).

## Discussion

### Sex-biased genes reflect different reproductive strategies in *V. montana*

Sexual dimorphism in dioecious plants constitutes a fundamental aspect of their life history, with influences extending beyond floral architecture to encompass the morphology and physiology of vegetative tissues, habitat preference or life history traits [64]. The expression of sex-biased genes is closely associated with sexual dimorphism in various plants and animals with separate sexes. For example, in *Ischnura elegans*, the up-regulation of the ommochrome pigmentation pathway in gynomorphic females was associated with ommochrome pigment accumulation underlying the genetically controlled female color polymorphism [65]. In fishes such as *Thalassoma bifasciatum*, *Salaria pavo*, and *Symphodus ocellatus*, transcriptomic profiles of none-gonadal tissues were found to more closely resemble phenotypic sex rather than anatomical sex [66–68]. In *T. pilosa*, female flower buds expressed a greater number of genes involved in defense against herbivorous insects and pathogens, which may be associated with the shorter lifespan, lower energy demands, and weaker chemical defense against herbivory in male flowers [7]. Therefore, investigating sex-biased gene expression is important for understanding the evolution of sexual dimorphism in dioecious plants [5].

Building upon this principle, our comparative transcriptome analysis reveals a clear functional divergence between the sexes in *V. montana*. Female-biased genes were primarily associated with photosynthesis, energy metabolism, hormone regulation and defense responses, suggesting a transcriptional program that supports the high resource demands and reproductive maintenance of female individuals [8, 35, 69]. In contrast, male-biased genes in flower buds were mainly enriched in transcription factor activity and transcriptional regulation, consistent with the intensive regulatory requirements of pollen development and male reproductive function [69, 70]. Although male-biased genes in leaves were enriched in signal transduction, energy metabolism, and defense-related pathways, their functional profiles partially overlapped with those of female-biased genes, indicating that both sexes may allocate resources toward maintaining vegetative performance while pursuing distinct reproductive strategies [71, 72].

Notably, the functional enrichment patterns of sex-biased genes identified in this study are highly consistent with previous reports in *V. montana*, despite the use of independent samples collected from different geographic locations and years [35]. Earlier studies similarly found that female-biased genes were preferentially involved in defense-related processes, whereas male-biased genes were mainly associated with pollen development [35]. The reproducibility of these findings across independent datasets suggests that sex-biased expression patterns in *V. montana* represent stable biological characteristics rather than transient environmental responses.

DNA methylation serves as a key mechanism governing spatiotemporal gene expression [26, 73]. In this study, the functional enrichment of DMR-mediated DEGs is broadly mirrored the transcriptional differences observed between the sexes. Female-associated pathways were mainly related to resource allocation, hormone signaling, and defense responses, whereas male-associated pathways were linked to transcriptional regulation and reproductive development. This concordance suggests that the establishment and maintenance of sex-biased expression programs in *V. montana* may be jointly regulated by genetic and DNA methylation mechanisms [30].

### Distinctive DNA methylation patterns in male and female tissues in *V. montana*

As an important epigenetic modification, DNA methylation plays a critical role in developmental transitions and cell fate determination in plants [74]. Developmental reprogramming of DNA methylation typically involves coordinated DNA demethylation and *de novo* methylation, processes that are particularly activate during reproductive development [75]. Previous studies have demonstrated that extensive methylation reprogramming occurs during pollen development, especially in transposable elements, imprinted loci, and epialleles, where it contributes to normal gametogenesis and reproductive success in species such as *Arabidopsis* and rice [75–78]. Compared with vegetative tissues, reproductive tissues generally undergo more extensive epigenetic remodeling to establish specialized developmental programs [79, 80]. Consistent with this pattern, we observed substantially more DMRs and DMR-mediated DEGs between female and male flower buds than between leaves. This finding suggests that DNA methylation dynamics are more closely associated with reproductive development than with vegetative growth in *V. montana*. Moreover, the distribution of DMRs differed markedly among sequence contexts, indicating that distinct methylation pathways may contribute to sex-specific developmental processes.

In plants, DNA methylation occurs in CG, CHG, and CHH sequence contexts, each characterized by different genomic distributions, maintenance mechanisms, and biological functions [21]. CG methylation is generally stable and heritable, whereas CHG and CHH methylation are considered more dynamic and responsive to developmental regulation [81–83]. Among the three contexts, CHH-type DMRs were overwhelmingly predominant in flower buds, with more than 99% of hypermethylated CHH-DMRs occurring in male flower buds and being concentrated in repetitive sequences. A similar increase in CHH methylation around transposable elements has been reported during reproductive cell differentiation in rice [75]. Given that suppression of transposable element activity is essential for maintaining genome stability during pollen development [84], the elevated CHH methylation observed in male flower buds may contribute to transposon silencing during male gametogenesis.

In addition, CHG hypermethylation showed distinct distribution patterns between the sexes, being preferentially enriched in repetitive regions in male flower buds but in gene bodies in female flower buds. Female flower buds generally exhibited fewer hypermethylated DMRs than male flower buds, with CG methylation representing the predominant hypermethylated context. These differences suggest that female and male flower buds may employ distinct epigenetic regulatory strategies during reproductive development. However, the functional consequences of these methylation patterns remain to be clarified through future studies integrating gene expression and functional validation data.

### Promoter DMR-mediated key TFs form tightly interconnected modules with hormone-responsive nodes and key floral development regulators in *V. montana*

In dioecious plants, TFs constitute central regulatory nodes controlling sex-specific developmental programs [85]. DNA methylation can contribute to the establishment of sex-biased expression patterns by modulating chromatin accessibility and transcriptional activity, particularly when occurring in promoter regions [26, 86]. This regulatory mechanism has been reported in several plant species. For example, loss of promoter methylation activates the *FLOWERING WAGENINGEN* (*FWA*) gene in *Arabidopsis*, resulting in delayed flowering, whereas sex-specific methylation of the sex-determining gene *PbRR9* in *Populus balsamifera* has been proposed to influence its transcription and contribute to sex differentiation [87, 88].

Consistent with these observations, we identified 11 key TFs whose expression was potentially associated with promoter DMRs and which also functioned as hub genes in co-expression networks. Several of these TFs are known regulators of flowering time and reproductive development. For example, *Vm101447* (*FRS5*) and *Vm222150* (*REM16*), *Vm109274* (*VRN1*) and *Vm124457* (*FAR1*) displayed male-biased or male-specific expression patterns. Their homologs in *Arabidopsis* are involved in the promotion of flowering [48, 49, 51], suggesting that they may contribute to the earlier floral differentiation and flowering observed in male *V. montana* individuals [35]. In contrast, the female-biased expression of *Vm126077* (*RVE6*), a photoperiod-responsive flowering regulator [50], indicates a potential role in coordinating female flower development. Likewise, the male-specific expression of *Vm208963* (*MYB35*), which is required for tapetum development and male fertility in asparagus [52, 89], suggests a conserved function in male reproductive development.

Plant hormones play pivotal roles in the establishment of sexual dimorphism in dioecious species [35]. Previous studies have demonstrated that jasmonic acid, salicylic acid, auxin, and brassinosteroid signaling pathways participate in sex-specific developmental processes. For example, the jasmonate coregulator MTB3 has been implicated in branch development in *Helianthus annuus* [90]. In *Litsea cubeba*, the accumulation of salicylic acid and the activation of downstream signaling regulators such as *LcTGA10* during the critical stage of female flower development induce programmed cell death in tapetal cells, leading to stamen abortion and the formation of unisexual female flowers [20, 91]. In grapevine, the promoter of the female-sterility candidate gene *YABBY3* contains binding motifs for the male-linked transcription factors, including BES1-INTERACTING MYC-LIKE1 (BIM1), a brassinosteroid signaling component involved in male fertility in *A. thaliana* [92]. Collectively, these findings indicated that hormone-responsive transcriptional networks are closely associated with the regulation of sexual differentiation.

Consistent with this notion, several promoter DMR-mediated TFs were involved in hormone-responsive pathways, including *Vm114640* (*MTB3*, a repressor of jasmonate signaling [56], *Vm103298* (*NF-YA3*), which participates in auxin responses [54], and two salicylic acid-responsive alleles *Vm103091* and *Vm203104* (*TGAL1*) [55]. Particularly noteworthy was the observation that the two *TGAL1* alleles, located on the Z and W chromosomes, exhibited opposite sex-biased expression patterns. Such divergence may reflect functional differentiation between homologous alleles during sex chromosome evolution and could be associated with sexually antagonistic selection [14]. Moreover, promoter DMRs in several TFs overlapped with cis-regulatory elements responsive to abscisic acid, auxin, or gibberellin. These findings suggest that DNA methylation may influence the sensitivity of hormone-responsive regulatory elements, thereby modulating TF activity during flower development.

Further support for this hypothesis was provided by co-expression network analysis. The floral organ polarity regulator *Vm104751* (*YAB2*) was strongly co-expressed with TFs involved in ethylene, gibberellin, abscisic acid, and jasmonate signaling, suggesting that it may function as an integrator of multiple hormonal pathways during floral organogenesis [60]. Similarly, *Vm103091* (*TGAL1*) and *Vm114640* (*MTB3*) exhibited strong co-expression relationships with several core developmental regulators, including *AGL11*, *SVP*, *NGA1*, and *DL* [93–96]. These interactions imply that hormone-responsive TFs may occupy central positions linking DNA methylation signals with downstream developmental programs.

Although the involvement of DNA methylation, TFs, and hormone signaling in sexual differentiation has been reported in several dioecious species, our study extends these observations by integrating methylome, transcriptome, and co-expression network analyses in *V. montana*. Rather than proposing an entirely novel mechanism, our results support a conserved epigenetic-transcriptional framework for sexual dimorphism while identifying species-specific regulatory components, including promoter DMR-mediated TFs and hormone-responsive co-expression modules. Together, these findings provide the first integrated view of the relationships among DNA methylation, transcriptional regulation, and hormone-responsive pathways in *V. montana*.

Collectively, our results support a species-specific regulatory framework in which promoter DNA methylation may influence the expression and hormone responsiveness of key TFs, thereby contributing to the establishment of sexual dimorphism in *V. montana*. It should be noted that the present study is primarily based on multi-omics analyses. The causal relationships implied in this model await further validation through targeted genetic and epigenetic experiments, such as gene editing and site-specific methylation manipulation.

## Conclusion

In this study, we systematically investigated sexual dimorphism in *V. montana* at the molecular level by integrating genome-wide DNA methylome and transcriptome analyses of flower buds and leaves. Our analyses included the functional characterization of sex-biased genes, the identification of DMR distribution patterns, and the discovery of key promoter DMR-mediated TFs and their potential regulatory relationships. Collectively, our results suggested that interactions among DNA methylation, transcriptional regulation, and hormone-responsive pathways may contribute to the establishment of sexual dimorphism in *V. montana*. These findings provide the first integrated view of these regulatory layers in *V. montana* and support a species-specific regulatory framework for understanding the epigenetic basis of sexual dimorphism in this economically important dioecious tree. However, the proposed framework is primarily based on multi-omics analyses. The causal roles of the identified DMRs, transcription factors, and methylation-regulated pathways remain to be validated through targeted functional experiments in vivo.

## Supplementary Information

Supplementary Material 1.

Supplementary Material 2.

## Data Availability

The RNA-seq data and DNA methylation sequencing data have been deposited in the Genome Sequence Archive (Genomics, Proteomics & Bioinformatics 2021) in the National Genomics Data Center (Nucleic Acids Res 2022), China National Center for Bioinformation / Beijing Institute Genomics, Chinese Academy of Sciences (GSA: CRA029599, CRA032624 and CRA056842) that are publicly accessible at https://ngdc.cncb.ac.cn/gsa.
