# Supplementary material for: Integrated methylome and transcriptome analysis provides insight into DNA methylation-mediated networks in sexual dimorphism of Vernicia montana
Source: BMC Plant Biol. 2026 Jun 26;26:1580. doi: 10.1186/s12870-026-09376-y (PMC13573456; doi:10.1186/s12870-026-09376-y)
Supplement: Supplementary file 1 [file 12870_2026_9376_MOESM1_ESM.docx]

Figure S1. Bar plot showing the number of HDMRs in CG, CHG, and CHH sequence contexts, comparing female and male floral buds and leaves.

Figure S2. Bar plot showing the number of DMR-mediated sex-biased genes in flower buds and leaves.

Figure S3. Functional annotation of gene co-expression modules. GO enrichment analysis of genes from the (A) yellow, (B) brown, (C) green, and (D) black modules derived from the co-expression network analysis.
